# Antibiotic Treatment in End-of-Life Cancer Patients—A Retrospective Observational Study at a Palliative Care Center in Sweden

**DOI:** 10.3390/cancers8090084

**Published:** 2016-09-06

**Authors:** Maria Helde-Frankling, Jenny Bergqvist, Peter Bergman, Linda Björkhem-Bergman

**Affiliations:** 1 ASIH Stockholm Södra, Långbro Park, Palliative Home Care and Hospice Ward, Bergtallsvägen 12, 125 59 Älvsjö, Sweden; maria.helde-frankling@sll.se; 2 Department of Laboratory Medicine, Division of Clinical Microbiology, Karolinska Institutet and Karolinska University Hospital, Huddinge, 141 86 Stockholm, Sweden; peter.bergman@ki.se; 3 Breast Centre, Department of Surgery, Capio St. Gorans Hospital, 112 81 Stockholm, Sweden; Jenny.Bergqvist@capiostgoran.se; 4 Department of Oncology/Pathology, Karolinska Institutet, 171 77 Stockholm, Sweden

**Keywords:** infections, antibiotics, palliative care, hospice care, cancer, CRP, immune system, vitamin D

## Abstract

*Background*: The aim of this study was to elucidate whether palliative cancer patients benefit from antibiotic treatment in the last two weeks of life when an infection is suspected. *Method*: We reviewed medical records from 160 deceased palliative cancer patients that had been included in previous studies on vitamin D and infections. Patients treated with antibiotics during the last two weeks of life were identified and net effects of treatment (symptom relief) and possible adverse events were extracted from medical records. *Results*: Seventy-nine patients (49%) had been treated with antibiotics during the last two weeks in life. In 37% (*n* = 29), the treatment resulted in evident symptom relief and among these 50% had a positive bacterial culture, 43% had a negative culture and in 7% no culture was taken. Among the patients with no or unknown effect of antibiotics, 50% had a positive culture. When the indication for antibiotic treatment was to avoid or treat sepsis, symptom relief was achieved in 50% of the patients (*n* = 19). Only 4% (*n* = 3) of the patients experienced adverse events of the treatment (diarrhea, nausea). *Conclusions*: Treating infections with antibiotics in the last weeks of life may improve the quality of life for palliative cancer patients, especially if sepsis is suspected or confirmed. According to our results, the beneficial effects outweigh the potentially negative outcomes.

## 1. Introduction

According to the WHO-definition of palliative care, early identification and assessment of physical symptoms in the palliative patient aim to prevent and relieve suffering. The overall goal is to improve quality of life in patients facing the problems associated with life-threatening illness. Therefore it is important that the side effects of any medical treatment do not outweigh possible beneficial effects.

In light of this basic principle the question whether suspected infections should be treated or not arises. Infections might give the patient bothering symptoms, such as fatigue, pain and discomfort. Especially symptoms of sepsis lead to distress. At the same time, non-restricted use of antibiotics in the dying patient may be questioned due to potential risks of allergic reactions, antibiotic-associated diarrhea and emergence of multi-drug resistant bacteria [1]. It is also possible that antibiotics may not be effective due to physiological alterations in the dying patient, such as decreased tissue perfusion, increased distribution volume and an impaired immune-system. Another practical implication of antibiotic-use in this patient-group is the increased need for use of invasive devices.

Infection in the dying patient is common. In an autopsy study from an inpatient hospice ward (*n* = 38) including both cancer and non-cancer patients, pneumonia was evident in 79% of cases and the leading cause of death in 44% of cases [2]. However, the clinical evidence for using or not using antibiotics in the end-of-life period is scarce and the results are divergent [3,4,5,6]. The frequency of antibiotic use in the dying cancer patient also varies greatly [1,7,8,9]. In a systematic review of studies of antimicrobial use in palliative care, the prevalence ranged from 4% to 84% [10].

There are no written guidelines regarding the use of antibiotics in the dying cancer patient and to treat or not to treat these patients needs ethical considerations. The difficulty of making sound treatment decisions in palliative patients have been addressed in several publications discussing such philosophical and ethical issues [11,12,13]. In a recent editorial, Furuno et al. argues that decisions on antibiotic use in hospice care should be made on a case-by-case basis and be consistent with the individuals goals of care [14].

In this study we set out to elucidate if cancer patients in the end-of-life situation experienced any benefit from antibiotic treatment when infections were suspected and whether antibiotic treatment had a positive or negative effect on patients’ symptoms. We wanted to test the hypothesis that antibiotics seldom do any harm in the end-of-life cancer patients and sometimes may improve quality of life. To this end, we retrospectively extracted data from medical records of 160 deceased cancer patients who had participated in previously performed studies related to vitamin D status and infections.

## 2. Methods

### 2.1. Study Cohort

To elucidate the effect of using antibiotics in the end of life we collected data from deceased cancer patients that had participated in two observational studies on vitamin D status and infections in palliative care; one study is completed and published [15], and one is still ongoing. The aim of the studies was to test the hypothesis that low vitamin D levels are associated with higher opioid dose, higher infectious burden and impaired quality of life in palliative cancer patients. One was a cross-sectional study to investigate the vitamin D levels and in the ongoing study patients with low vitamin D-levels are supplemented with vitamin D. Palliative cancer patients were recruited from ASIH Stockholm Södra, Långbro Park Advanced Palliative Home Care Team and Hospice Ward. The patients were recruited consecutively, i.e., they did not have any specific infections at inclusion and patients with any type of cancer were included. In these two studies 163 patients had deceased at the end of April 2016. Three patients had moved from Stockholm or had changed care-givers, which precluded access to the medical records (missing data). In total, *n* = 160 patients were included in the final analysis.

### 2.2. Data Extraction

We identified all patients with any antibiotic treatment in their last two weeks of life. For these patients, data were collected from the medical records regarding type of infection, microbiological cultures, pathogens, type of antibiotics and indication of treatment divided into five groups: (1) treatment or prevention of sepsis; (2) respiratory tract symptoms; (3) urinary tract symptoms; (4) GI-tract symptoms; (5) skin infections. In general, when no obvious infectious focus was evident but the patient had fever, elevated CRP and an impaired general condition the indication for antibiotic use was “prevention of sepsis”. Data on positive effects of treatment, such as different forms of symptom relief or increased quality of life stated in the medical or nursing records was collected. Examples of “positive effects” were reduced fatigue, resolution of fever or that the patient gained energy to do things of their choice that were not possible before antibiotic treatment was initiated. If no positive effects were mentioned in the medical record the effect was classified as “no effect” or “unknown”. In addition, possible adverse reactions to the antibiotic treatment stated in the medical records were also collected. The assessment of the effects of antibiotics was performed by two independent reviewers (MHF and LBB). If there were divergent opinions the case was discussed and a consensus decision was taken.

Demographic data regarding age, sex and cancer diagnosis had already been collected previously. The latest CRP and albumin levels were collected. No values were older than 3 weeks before death. Vitamin D was measured as 25-hydroxyvitamin D and only levels measured less than 60 days before death was included in the analysis, since the half-life of 25-hydroxvitamin D is 3–4 weeks and values older than 2 month were considered to be non-representative of the current vitamin D status.

### 2.3. Statistical Analysis

**Figure 1:**
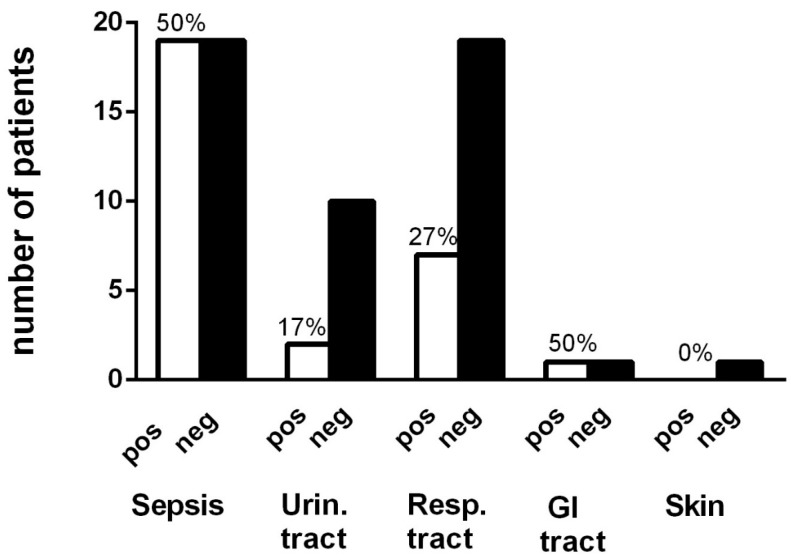
Effect of antibiotics at different infection indications in end-of-life cancer patients. Positive effect (pos) or unknown/no effect (neg) are presented. Sepsis means to treat or avoid sepsis. Percentage of positive effect of all treatment in that specific infections indication is stated. When comparing the outcome of treatment of sepsis and urinary tract infections it was more likely to have a positive effect when treating sepsis, OR 5.0; 95% CI 0.96–25.9 (*p* = 0.05).

Statistical analyses were performed using Graph Pad Prism vs. 6.0. Since some of the data did not show a Gaussian distribution we present median levels and ranges. When comparing demography parameters, CRP, albumin and 25-hydroxyvitamin D levels, Mann-Whitney U-test was used. When comparing the distribution of “positive”, “negative” or “no culture” and “antibiotic effects” in different cancer forms, Chi^2^-test was used. When comparing “positive” or “no effect of antibiotic treatment” in sepsis or urinary tract infections, Fisher’s exact test was used (Figure 1).

### 2.4. Ethic Statement

The original studies were approved by the local Ethical Committee at Karolinska Institutet, Stockholm, Sweden (Dnr: 2014/455-31/4 and 2015/776-31) and was performed in accordance with the declaration of Helsinki. Written informed consent was obtained from all patients before inclusion in the original study which enabled us to follow the outcome of the patients, as registered in the medical records regarding infections, pain and quality of life.

## 3. Results

### 3.1. Demography, Antibiotics and Infections

**Figure 2:**
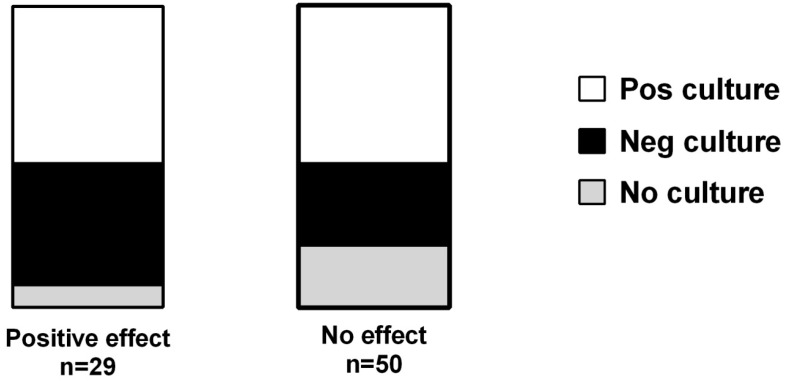
Distribution of number of positive and negative cultures among end-of-life cancer patients in patients who had positive (Pos effect) or unknown/no effect (No effect) of antibiotic treatment. There was no statistically significant difference between the distribution of positive and negative cultures between the groups (Chi^2^-test, *p* = 0.22).

The demography of all patients is shown in Table 1. Of the 160 patients in this cohort, 79 (48%) had been treated with any antibiotic during the last week in life. The mean CRP-values were significantly higher in antibiotic treated patients than in non-treated patients (*p* < 0.001). There were no significant differences in albumin levels, age or gender distribution between the two groups (Table 1). Of the 79 patients who were treated, evident symptom relief was achieved in 37% (*n* = 29). Notably, only 4% (*n* = 3) of the patients experienced any adverse event (diarrhea in two cases, nausea in one case) (Table 1). Among the patients with a positive effect of the treatment, only 50% had a positive culture, 43% had a negative culture and in 7% no culture had been taken (Figure 2). Among the patients with no or unknown effect of antibiotics, 50% had a positive culture, 30% had a negative culture and in 20% no culture had been taken (Figure 2). There was no statistically significant difference between the distribution of positive and negative cultures between the groups.

When “prevention or treatment of sepsis” was the indication for antibiotic treatment, a statistically significant association with symptom relief was observed in 50% of the patients (*n* = 19). In contrast, when a urinary tract infection was suspected and used as an indication for antibiotic treatment only 17% (*n* = 2) experienced symptom relief (Fishers exact test showed OR 5.0; 95% CI 0.96–25.9; *p* = 0.05) (Figure 1).

The most common antibiotic used was ceftriaxone (27%), followed by piperacillin/tazobactam (20%) and cefotaxime (13%). The majority had a parental route of administration (77%) and the remainder had oral antibiotics (23%).

Bacterial cultures were taken in 67 patients and the different pathogens found in the 41 patients with positive bacterial cultures are presented in Table 2. *Staphylococcus aureus* was the most common pathogen found. Two of the bacterial cultures revealed *E. coli* expressing extended spectrum beta-lactamase (ESBL). There was no positive culture for methicillin resistant *S. aureus* (MRSA).

### 3.2. Type of Cancer

To study if the use or response to antibiotics varied between different cancer types we grouped the patients into 14 different cancer types presented in Table 3. 

The most common cancer was lung cancer, followed by gastrointestinal cancer and breast cancer. There was no statistically significant difference between the different cancer forms regarding treatment with antibiotics or whether the antibiotic use had any beneficial effect on symptoms or not (Chi^2^ test, *p* = 0.99 and *p* = 0.73). The lowest effect of antibiotics was found in prostate cancer where only 12% had a positive effect of the treatment. However, when comparing these patients with the rest of the cohort no statistically significant difference was observed (Fishers exact test, *p* = 0.25).

### 3.3. Vitamin D and Effect of Antibiotics

Vitamin D status is important for host immunity against various infections [16,17,18] and has also been suggested to play a role in the quality of life of palliative cancer patients [15,19]. Thus, we evaluated whether the vitamin D status of the patients was related to antibiotic-use or with response to treatment. Representative vitamin D levels were available for 123 patients, i.e., levels measured less than 60 days before death (Table 1). There was no association found between vitamin D levels and antibiotic use (*p* = 0.20) or with response to treatment (*p* = 0.32). In the cohort there were only 17 patients with “sufficient” vitamin D levels, defined as 25-hydroxyvitamin D levels > 75 nmol/L. Of these 17 patients six received antibiotics (35%) compared to 55% for those with lower vitamin D-levels (*p* = 0.19). Among the six patients with sufficient vitamin D levels, four responded (67%) to antibiotics and two (33%) did not respond, compared to 33% responders and 67% non-responders among those with insufficient levels. However, this difference did not reach statistical significance, as Fischer’s exact test showed *p* = 0.17.

## 4. Discussion

Here we show that antibiotic treatment resulted in improved symptoms in 37% of the patients in this cohort of palliative cancer patients. Interestingly, also in cases with negative cultures the administration of antibiotics appeared to have beneficial effects and, importantly, the prevalence of adverse events was not common (<4%).

The clinical evidence for using or not using antibiotics in the end-of-life period is scarce, and there are no written guidelines [20]. Results from previous studies are divergent. Our findings are in line with the study by Vitetta et al. (*n* = 102), where it was shown that 40% of terminally ill patients achieved symptom control after antibiotic treatment [6]. Furthermore, a nationwide survey evaluated 282 patients treated with antibiotics in both hospice and outpatient settings and 56% of the patients were evaluated as having “good” or “very good” clinical effect of antibiotics [5]. In a university hospital with a mean duration of 31 days between admission and death, a total of 84% of patients were treated with antibiotics during admission, and 63% at time of death. Symptomatic improvement was achieved in only 15% of the patients, but 48% of the patients with fever experienced an improvement [4]. Another single-centre retrospective study from an inpatient hospice ward registered antibiotic use in 85% of febrile episodes in a population with a median survival of 15 days after initiation of antibiotic treatment. Resolution of fever was achieved in 54% of patients in the treatment group and in 2% of patients not treated with antibiotics (*p* = 0.004) [3].

In contrast to our results a study including 255 patients showed that the use of antibiotics was associated with symptom relief for urinary tract infections, but it was less effective for other infections including bacteremia and sepsis [21]. However, symptom relief from urinary tract infections might be underestimated in our cohort since this is more seldom documented in the records than relief from septic symptoms.

Also in non-cancer patients there is controversy regarding the effect of antibiotic treatment in the last days and weeks of life, since data from different epidemiological studies vary. One study including 559 palliative non-cancer patients with pneumonia revealed that antibiotic treatment decreased discomfort even when death was imminent [22]. In contrast, another study (*n* = 225) showed that antibiotic treatment did not improve symptoms in terminally ill patients with pneumonia although the survival time was increased [23]. It is noteworthy that serious infections may produce sedation leading to a peaceful death, whereas the administration of antibiotics can prolong the process of dying and thereby paradoxically increase the experience of suffering [24].

The conflicting results from previous epidemiological studies might reflect how difficult it is to assess if a patient is dying or suffering from a temporary deterioration due to an infection. In addition to that, the immune system in terminally ill patients is probably impaired and the effectiveness of antibiotics might be reduced. For example, it has been shown that there is a dysregulation of innate immune responses in the aging body leading to more inflammatory response, sometimes called “inflammaging” [25]. Notably, CRP increases in the end-of-life in cancer patients also in the absence of infections. Indeed, elevated CRP has been shown to be a good prognostic marker for estimation of the time of death [15,26]. However, this increase in CRP is often misinterpreted as an infection and treatment is initiated—although bacterial cultures later are found to be negative and the patient dies.

Our study reports a large proportion of intravenously administered antibiotics (77%), reflecting current practice at our site, where a majority of patients have an intravenous line (picc-line or subcutaneous venous access device). The practice of using intravenously administered drugs in the end-of-life patients might vary largely between different countries but is common in palliative wards in Sweden.

*S. aureus* and *E. coli* were the two most common pathogens in our patient cohort, well in line with previous studies [4,5,21,27]. Treatment against *S. aureus* was only associated with symptom relief when the bacterium was found in synovial fluid (one patient), suggesting that *S. aureus* often is a colonizer and not always causes a clinically relevant infection. Likewise, antibiotic treatment of bacteria found in various wound cultures was not associated with improvement of symptoms in this patient-group. However, skin cultures are not specific for active infection. In addition, it should be noted that positive a urine culture does not necessary indicate urinary tract infection, since asymptomatic bacteriuria is common among frail patients.

Notably, there was a high frequency of *S. epidermidis* in blood cultures (7/15), raising suspicion of contamination, but it might also reflect the high prevalence of invasive devices in the patients in this particular cohort. Multidrug-resistant organisms (MDROs) were found in 2/41 cultures (ESBL). This correlates well with the comparatively low frequency of MDROs in the Swedish population (http://ecdc.europa.eu/en/healthtopics/antimicrobial_resistance/database) and differs from results from other cohorts where MDROs were found in ¼ of cultures in patients who died during hospital admission [1].

In our study 48% of terminally ill cancer patients were treated with antibiotics in the last week of life, placing our single center experience near the middle of the range (4%–84%) of the prevalence of antimicrobial treatment as shown in a systematic review including studies from different countries [10]. Differences in patients’, families’ and health care professionals’ attitudes [11,12,13] as well as differences in the organization and financing of health care systems might explain the diverging results regarding the frequency of antibiotics treatment near the end of life. Comparing US and Asian experiences demonstrate large differences in current practice. A nationwide US survey showed that 14% of patients admitted to hospice with cancer as primary diagnosis were treated with antibiotics in the last week of life [28]. In two different US cohorts from academic hospitals, cancer patients who transitioned to comfort focused care remained on antimicrobial treatment in 20% and 35% of cases [8,9]. In contrast, a small study from a Chinese palliative care unit showed that 18/21 patients with hematological malignancies received i.v. antibiotics during the last week of life [7]. In a Korean cohort of deceased patients (75.6% with solid tumors) who had been treated in general medicine wards in acute care hospitals, 87.5% had received antibiotic treatment lasting more than 24 h [1].

There a several limitations of this study. Most importantly, this is a retrospective study where our assessment of the potential response to antibiotics is based solely on the medical- and nursing records. Thus, cases with symptom relief may be underestimated since this is not always mentioned in the records. This might explain the divergence between the positive effects for septic symptoms in contrast to urinary tract infections. It should also be mentioned that the true source of infection is often hard to determine. For example, a positive urine or sputum culture does not necessarily indicate urinary tract infection or pneumonia, respectively. Thus, data on the source of infections must be interpreted with caution. Another limitation is the relatively small sample size. Especially, the few cases for each cancer type make this subgroup analysis less reliable. On the other hand, studies on the effect of antibiotics in the end-of-life cancer patients are scarce and this study supports the hypothesis that antibiotics seldom do any harm in these patients. This is also the first study where the response to antibiotics in the dying patient is correlated with vitamin D status of the patients. Our findings show that there is no clear association between vitamin D status and antibiotic response in the dying patient, although there was a trend towards a better outcome for patients with sufficient vitamin D levels (*p* = 0.17).

## 5. Conclusions

In conclusion, treating infections in the last weeks of life may contribute to improving the quality of life for palliative cancer-patients, especially if sepsis is suspected or confirmed. According to our results, beneficial effects are generally more common than harmful effects. However, the efficacy of antibiotics might not be optimal in the dying patient. The decision to treat or not to treat suspected infection in the terminally ill patient is complex and requires an individual approach and ethical considerations. If possible the decision should preferably be taken together with the patient and the relatives, which is a recommendation that aligns with recent publications in the field [14].

## Figures and Tables

**Table 1:** Demographic data of the study cohort of 160 end-of-life cancer patients. Effect of antibiotic treatment; positive effect (Pos effect) or unknown/no effect (No effect); is presented. Reference range according to Clinical Chemistry Dept. of Karolinska University Hospital: CRP <3 mg/L, Vit. D 75–250 nmol/L, Albumin 36–48 nmol/L. * CRP-levels among the antibiotic treated patients were significantly higher than for non-treated patients, *p* < 0.05. There were no other statistical significant differences between the groups. NA = not applicable.

	Age Median (Range)	Men % (*n*)	CRP mg/L Median (Range)	Vit. D nmol/L Median (Range)	Albumin g/L Median (Range)	Adverse Event % (*n*)	Culture Taken % (*n*)	Pos Culture % (*n*)
All (*n* = 160)	71 (18–95)	43% (69)	61 (1–597)	36 (8–133)	24 (11–39)	NA	NA	NA
No Antibiotics (*n* = 81)	72 (32–95)	42% (34)	44 * (1–283)	39 (8–133)	25 (16–39)	NA	NA	NA
Antibiotics (*n* = 79)	68 (18–90)	44% (35)	124 * (1–597)	33 (8–120)	22 (11–37)	3.8% (3)	85% (67)	52% (41)
Pos effect (*n* = 29)	69 (36–90)	34% (10)	76 (7–319)	37 (10–112)	22 (14–31)	3.4% (1)	93% (27)	52% (15)
No effect (*n* = 50)	68 (18–86)	50% (25)	141 (1–597)	32 (8–120)	22 (11–37)	4% (2)	80% (40)	52% (26)

**Table 2:** Pathogens in the positive cultures (*n* = 41 patients).

Cultures	Pos Effect, *n* = 15 (15 Cultures)	No Effect, *n* = 26 (32 Cultures)
Blood	7 *S. epidermidis* 3 *Enterococcus species* 1 *Corynebacterium species* 1 *Klebsiella species* 1 *S. marscecens* 1	8 *S. epidermidis* 4 *Enterococcus species* 1 *Enterobacter species* 1 *S. aureus* 1 *Klebsiella species* 1
Urine	4 *E. coli* 2 *Klebsiella species* 2	14 E. coli 7 *Klebsiella species* 1 *S. aureus* 1 *Enterococcus species* 3 *Enterobacter species* 1 *Legionella species* 1
Skin	0	5 *S. aureus* 4 *E. faecialis* 1
Sputum	1 *Bacillus species* 1	3 *S. aureus* 3
Feces	1 *C. difficile* 1	0
Nasopharynx	0	2 *M. catharralis* 1 *S. aureus* 1
Synovial fluid	1 *S. aureus* 1	0
Pleura fluid	1 *S. epidermidis* 1	0

**Table 3:** Type of cancer in the whole cohort and among those with antibiotic treatment. In column 3 and 4 patients with positive effect (Pos effect) or unknown/no effect (No effect) of the antibiotic treatment is presented and percentage intends of those with that specific cancer form receiving antibiotics (column 2). NA = not applicable.

Type of Cancer	All (*n* = 160)	Antibiotics (*n* = 79)	Pos Effect (*n* = 29)	No Effect (*n* = 50)
Brain tumor	4	1	0	1 100%
Breast cancer	19	10	3 30%	7 70%
Gynecological	16	9	4 44%	5 56%
GI-cancer	29	13	5 38%	8 62%
Gallbladder	6	3	2 67%	1 33%
Head-Neck cancer	8	4	2 50%	2 50%
Hematologic mal.	8	3	2 67%	1 33%
Kidney	3	2	1 50%	1 50%
Lung cancer	32	19	6 32%	13 68%
Malignant Melanoma	3	0	NA	NA
Mesotelioma	2	1	1 100%	0
Pancreas	14	6	2 33%	4 67%
Prostate cancer	13	8	1 12%	7 88%
Other	3	0	NA	NA
